# Engineering viable foot-and-mouth disease viruses with increased acid stability facilitate the development of improved vaccines

**DOI:** 10.1007/s00253-019-10280-9

**Published:** 2020-01-03

**Authors:** Hong Yuan, Pinghua Li, Huifang Bao, Pu Sun, Xingwen Bai, Qifeng Bai, Na Li, Xueqing Ma, Yimei Cao, Yuanfang Fu, Kun Li, Jing Zhang, Dong Li, Yingli Chen, Jie Zhang, Zengjun Lu, Zaixin Liu

**Affiliations:** 1 State Key Laboratory of Veterinary Etiological Biology, OIE/China Foot-and-Mouth Disease Reference Laboratory, Lanzhou Veterinary Research Institute, Chinese Academy of Agricultural Sciences, , No. 1 Xujiaping, Yanchangbao, Lanzhou, 730046 Gansu People’s Republic of China; 2 Key Laboratory of Preclinical Study for New Drugs of Gansu Province, School of Basic Medical Sciences, Lanzhou University, Lanzhou, 730046 Gansu People’s Republic of China

**Keywords:** FMDV, Vaccines, Acid stability, Substitution

## Abstract

Foot-and-mouth disease virus (FMDV), the most acid-unstable virus among *picornaviruses*, tends to disassemble into pentamers at pH values slightly below neutrality. However, the structural integrity of intact virion is one of the most important factors that influence the induction of a protective antibody response. Thus, improving the acid stability of FMDV is required for the efficacy of vaccine preparations. According to the previous studies, a single substitution or double amino acid substitutions (VP1 N17D, VP2 H145Y, VP2 D86H, VP3 H142D, VP3 H142G, and VP1 N17D + VP2 H145Y) in the capsid were introduced into the full-length infectious clone of type O FMDV vaccine strain O/HN/CHN/93 to develop seed FMDV with improved acid stability. After the transfection into BSR/T7 cells of constructed plasmids, substitution VP1 N17D or VP2 D86H resulted in viable and genetically stable FMDVs, respectively. However, substitution VP2 H145Y or VP1 N17D + VP2 H145Y showed reverse mutation and additional mutations, and substitution VP3 H141G or VP3 H141D prevented viral viability. We found that substitution VP1 N17D or VP2 D86H could confer increased acid resistance, alkali stability, and thermostability on FMDV O/HN/CHN/93, whereas substitution VP1 N17D was observed to lead to a decreased replication ability in BHK-21 cells and mildly impaired virulence in suckling mice. In contrast, substitution VP2 D86H had no negative effect on viral infectivity. These results indicated that the mutant rD86H carrying substitution VP2 D86H firstly reported by us could be more adequate for the development of inactivated FMD vaccines with enhanced acid stability.

## Introduction

Foot-and-mouth disease (FMD) is a highly infectious vesicular disease of domestic and wild cloven-hoofed animals. This disease remains enzootic in many regions of the world and causes severe economic losses worldwide (Grubman and Baxt 2004; Sobrino and Domingo 2001). Its causative agent is the FMD virus (FMDV), a member of the genus *Aphthovirus* in the family *Picornaviridae*. In areas where FMDV is endemic, disease control is carried out predominantly by vaccination. Currently, chemically inactivated vaccine is widely applied to prevent and control FMD, and the recombinant empty capsid vaccine shows promising future for its safety (Doel 2003; Rodriguez and Grubman 2009). However, both the FMDV particles and the recombinant empty capsids are sensitive to acidic condition, severely influencing the shelf-life of the vaccines and affecting the induction of protective immunity (Brown and Cartwright 1961; Doel and Chong 1982). Thus, improving the acid stability of virions or empty capsids is required.

FMDV genome includes a single-stranded, positive-sense RNA molecule of approximately 8500 nucleotides (Grubman et al. 1984). FMDV is a non-enveloped particle with icosahedral symmetry. The virus capsid contains 60 copies each of the four structural proteins (VP1, VP2, VP3, and VP4). During the capsid assembly, one copy of four proteins folds into a protomer; then, five copies of protomer compose a pentamer; at last, 12 pentamers self-assemble to form a capsid shell (Acharya et al. 1989; Vasquez et al. 1979). Among all members in the family of *Picornaviridae*, the FMDV particle is highly acid labile due to the rapid dissociation of capsid into pentameric subunits at pH slightly below neutrality (Newman et al. 1973). This acid sensitivity property of FMDV is needed for the uncoating activity in the early endosome of host cell, but during the process of vaccine preparation and storage, the acid stability of virion is demanded (Huotari and Helenius 2011; O'Donnell et al. 2005). Therefore, it is important to keep a balance between acid stability and acid sensitivity to maintain the intact virion during manufacture of inactivated vaccine without impairing the viral uncoating and replication within the infected cells. The stability of FMDV capsid depends on a variety of electrostatic interactions between the capsid subunits and nucleic acid. These interactions are easily affected by the alteration of pH and temperature in the environment, leading to the dissociation of the capsid (Caridi et al. 2015; Curry et al. 1995; Martin-Acebes et al. 2010; Mateo et al. 2008; Rincon et al. 2014; van Vlijmen et al. 1998).

Recent studies have shown that a single amino acid substitution in the virus capsid could increase acid stability and sensitivity of FMDV. To date, a number of amino acid residue substitutions responsible for the acid sensitivity of FMDV have been identified (Biswal et al. 2016; Caridi et al. 2015; Liang et al. 2014; Martin-Acebes et al. 2010; Martin-Acebes et al. 2011; Vazquez-Calvo et al. 2014; Wang et al. 2014; Yuan et al. 2017). According to the reported substitutions affecting virion acid stability (Yuan et al. 2017), part of them were chosen to improve the acid stability of FMDV O/HN/CHN/93 in this study. The substitution VP1 N17D could increase the acid resistance of type C, O, and Asia I FMDVs (Liang et al. 2014; Martin-Acebes et al. 2011; Wang et al. 2014). The single substitution VP2 H145Y induces an increase in resistance of C-S8c1 to acidic treatment (Wang et al. 2014). In addition, the double replacements VP1 N17D + VP2 H145Y confer marked stability with pH_50_ (pH value that causes a 50% loss of infectivity) of 5.4 for C-S8c1 (Vazquez-Calvo et al. 2014). Earlier, residue H141 in VP3 (type A FMDV) has been reported to modulate the acid sensitivity of FMDV because of the electrostatic interactions across the inter-pentameric interfaces induced by protonated His (Ellard et al. 1999; van Vlijmen et al. 1998). Recently, residue VP3 H142 (type O FMDV) has been separately substituted to Arg (R), Phe (F), Ala (A), and Asp (D), but only VP3 H142D mutation resulted in the generation of mutant FMDV with increased acid resistance (Biswal et al. 2016). Now, it is still not clear whether the side chain or the charge of residue VP3 H142 is the critical, so we modified this residue separately to acidic Asp (D) or Gly (G) without side chain. Amino acid residue substitution VP2 D86A and VP2 D86H have been observed in type O FMDV mutants with increased acid stability, but substitution VP2 D86A is not responsible for the acid stability phenotype and it is still unknown that weather substitution VP2 D86H could result in an increase in acid resistance (Liang et al. 2014). Therefore, the influence of VP2 D86H substitution on FMDV acid stability was assessed here.

In this work, to produce genetically modified seed virions with increased acid stability, single or double substitutions (VP1 N17D, VP2 H145Y, VP2 D86H, VP3 H141D, VP3 H141G, and VP1 N17D + VP2 H145Y) were introduced into infectious clone pOFS to generate FMDVs with acid stability. As few mutations could be accepted by virus without affecting its infectivity, we also characterized the biological and biophysical properties of rescued FMDVs to screen out acid-resistant mutants with normal or slightly decreased infectivity (Carreira and Mateu 2006; Mateo et al. 2003; Reguera et al. 2004). The results showed that both substitution VP1 N17D and VP2 D86H could independently confer increased acid resistance, alkali resistance, and thermostability on FMDV O/HN/CHN/93. However, substitution VP1 N17D was observed to lead to a decreased replication capacity in BHK-21 cells and mildly reduced virulence in suckling mice. In contrast, substitution VP2 D86H had no negative effect on viral infectivity, indicating its potential application in improved vaccines. To sum up, we firstly reported that the mutant rD86H carrying substitution VP2 D86H could be more adequate for the development of inactivated FMD vaccines with enhanced acid stability.

## Materials and methods

### Cells, virus, plasmids, and virus titrations

The origin and culture procedures for BHK-21 and BSR-T7/5 cells have been described (Buchholz et al. 1999; Li et al. 2012a; Rieder et al. 1993). Plasmid pOFS contains the complete genome of FMDV O/HN/CHA/93 (Li et al. 2012b). Virus titration is determined by calculating the 50% tissue culture infectious dose per mL (TCID_50_/mL).

### Construction of the full-length plasmids containing site-directed mutagenesis and transfection of BSR/T7 cells

According to the reported substitutions responsible for the resistance to acidic pH, substitutions VP1 N17D, VP2 H145Y, VP2 D86H, VP3 H141D, VP3 H141G, and VP1 N17D + VP2 H145Y were separately introduced into the infectious clone pOFS using site-directed mutagenesis. Finally, the newly constructed plasmids were termed as pN1017D, pH2145Y, pN1017D + H2145Y, pD2086H, pH3141D, and pH3141G. The engineered plasmids (2 μg) linearized by *Not* I were used to transfect BSR/T7 cells using Lipofectamine™ 2000. After appearance of apparent cytopathic effect (CPE), recovered viruses were harvested. To analyze the genetic stability of substitutions during virus replication, the sequences of complete capsid gene of rescued viruses were amplified to identify the substitutions after 10 rounds of infection of BHK-21 cells.

### Acid sensitivity analysis

Acid-induced inactivation assay was conducted according to the published procedures (Caridi et al. 2015; Martin-Acebes et al. 2010). Equal numbers of virus particles containing 2 × 10^6^ PFUs were mixed with 300 μL of PBS solutions (50 mmol/L NaH_2_PO_4_ and 140 mmol/L NaCl) of different pH values (6.0, 6.2, 6.4, 6.6, 6.8, and 7.4) for 30 min at room temperature. Then, the mixture was neutralized with 100 μL of 1 M Tris (pH 7.4), and the recovered viruses were determined by plaque assay on BHK-21 cells. Infectivity was calculated as the percentage of PFU recovered at each different pH relative to that obtained at pH 7.4. The pH_50_ values of all viruses were calculated and the statistically significant differences were analyzed using a one-way ANOVA.

The capsid dissociation induced by the acid treatment was measured. The virus strains were chemically inactivated by BEI at 30 °C for 28 h, and subsequently, 600 μL of inactivated viruses were mixed with 300-μL PBS solutions (50 mmol/L NaH_2_PO_4_ and 140 mmol/L NaCl) of different pH values (6.0, 6.2, 6.4, 6.6, 6.8, and 7.4) for 30 min at room temperature. The solution was neutralized by adding 100 μL of 1 M Tris (pH 7.4). The remaining 146S in the samples was analyzed by the size-exclusion high-performance liquid chromatography (SE-HPLC) method which could automatically and quickly read out the contents of 146S in the samples by reference to the standard curve (Yang et al. 2015). The percentage of intact virions disposed at different pH values relative to those acquired at pH 7.4 was determined.

### Biological characteristics of rescued FMDV mutants

Plaque-forming assay of the rescued viruses was performed in duplicate. BHK-21 cells in 6-well plates were infected with the mutants and parental virus for 1 h, followed by the addition of 2 mL overlay. After incubation at 37 °C for 45 h, the cells were stained with 0.2% crystal violet and the plaque phenotype was observed.

The replication ability of different mutants was measured by one-step growth curve analysis. BHK-21 cells were infected with mutant viruses and WT virus at a multiplicity of infection (MOI) of 1 at 37 °C for 1 h. After removement of virus supernatant, the cells were washed with PBS (pH 7.4) and supplemented with DMEM culture media. Cell samples were harvested at 4, 8, 12, and 20 h post-infection. Virus titers were measured by the TCID_50_ assay.

Virulence analysis of different mutants was also evaluated in suckling mice. Four groups of 1-day-old suckling mice (5 per group) were subcutaneously inoculated with different TCID_50_ doses (100 μL) of mutant and parental viruses. As a control, an equal amount of neutral PBS was injected into another group of mice. The percentage of surviving mice was calculated 6 days post inoculation.

### Alkali-induced inactivation assay

The modified viruses (2 × 10^6^ PFUs/mL) in 20 μL were incubated with 300 μL of alkaline PBS (varying in pH from 8.8 to 9.8) for 30 min at room temperature, and then, the pH was neutralized with 200 μL of 1 M Tris (pH 7.4). The surviving viruses were titrated by plaque-forming assay.

### Thermal inactivation assay

The thermostability of mutant viruses was determined following the previously published protocol (Mateo et al. 2003; Mateo et al. 2008). The viruses were incubated at 42 °C for 20, 40, or 60 min, and the titer of the remaining viruses at each time point was determined by the plaque-forming assay.

### Effect of acid treatment at different ionic strengths on inactivation

Equal numbers of virus particles (2 × 10^6^ PFUs/mL) were mixed with 300 μL of PBS buffer (ranging from pH 6.0 to 7.4) and 200-μL NaCl solutions (150 mM or 1 M) for 30 min at temperature. After neutralization, the plaque assay was used to determine the titers of remaining virions.

### Model construction and analysis

The crystal structure of FMDV capsid, which contains four particles (VP1, VP2, VP3, and VP4), was extracted from the PDB database (PDB ID: 1FOD) (Yang et al. 2015). The accurate model of FMDV was constructed based on this known structure using the TCL programming files. The pentamer formed by five monomers of VP1, VP2, VP3, and VP4 was obtained from the model of FMDV via VMD1.9.2 software (Humphrey et al. 1996). The mutations of VP1 N17D, VP2 H145Y, VP2 D86H, VP2 D86A, VP3 H141G, and VP3 H141D were built by the open-source Pymol-v1.7.6.0, which has been compiled by us. The energy minimization of these mutants was performed by UCSF Chimera1.10.2 (Pettersen et al. 2004). The steps of steepest descent and conjugate gradient were, respectively, set to 1000 and 500. The step sizes of steepest descent and conjugate gradient were both set to 0.02 Å. To calculate the hydrogen bond of residues in the FMDV, the angle and distance between two molecules were set less than 3.0 Å and 35 °C, respectively. The representational figures were generated by VMD1.9.2 (Humphrey et al. 1996) and open-source Pymol-v1.7.6.0.

### Guinea pig immunization

Female guinea pigs (250 to 350 g) were randomly divided into five groups (5 per group). Four groups of guinea pigs were intramuscularly inoculated with 1.0 μg of purified mutants rN17D, rD86H, rN17D2, or parental FMDV O/HN/CHN/93. As control, one group of guinea pigs was immunized with an equal volume of PBS. Serum samples of guinea pigs were collected from heart on days 0, 14, 21, 28, and 35 post-vaccination.

### Virus neutralization assay

According to the OIE Manual of Standards, neutralizing antibody titer of guinea pig serum was determined by the micro-neutralization test. Briefly, 50 μL of FMDV O/HN/CHN/93 containing 100 TCID_50_ was mixed with 50 μL of two-fold serial diluted serum in a 96-well microplate (Corning, USA). After incubation for 1 h at 37 °C, 50 μL of BHK-21 cells were added to each well. The cells were fixed and stained with 10% methanol and 0.05% methylene blue solutions after incubation for 72 h. At last, the neutralizing titer (NT_50_) was calculated.

## Results

### Production of FMDV mutants and genetic stability analysis

The six linearized full-length cDNA clones (pN1017D, pH2145Y, pN1017D/H2145Y, pD2086H, pH3141D, and pH3141G) carrying modifications were transfected into BSR/T7 cells expressing T7RNA polymerase. However, only four constructions (pN1017D, pH2145Y, pN1017D/H2145Y, pD2086H) produced infectious FMDVs. The H3141D or H3141G mutation blocked viral viability. The capsid coding regions of four rescued viruses (rN17D, rD86H, rH145Y, and rN17D/H145Y) were sequenced and compared to that of nonmutated O/HN/CHN/93 virus. Unexpectedly, only rN17D and rD86H mutants maintained the introduced substitution without further nucleotide mutation after 10 passages in BHK-21 cells. For virus rN17D/H145Y, H-to-Y replacement was reversed to the parental sequence and substitution N-to-D was still retained. The H-to-Y mutation in virus rH145Y also showed reversion. Moreover, other additional mutations (VP3 A95G, VP3 Q96P, VP3 Q220H, and VP1 R38K) were found in both viruses rH145Y and rN17D/H145Y. Ultimately, two genetically stable FMDVs (rN17D and rD86H) were finally generated. In order to explore whether the additional mutations VP3 A95G, VP3 Q96P, VP3 Q22H, and VP1 R38K together had a compensating effect on VP1 N17D, the property of virus rN17D/H145Y (rN17D2) was further studied.

### pH stability of the rescued FMDVs

**Fig. 1:**
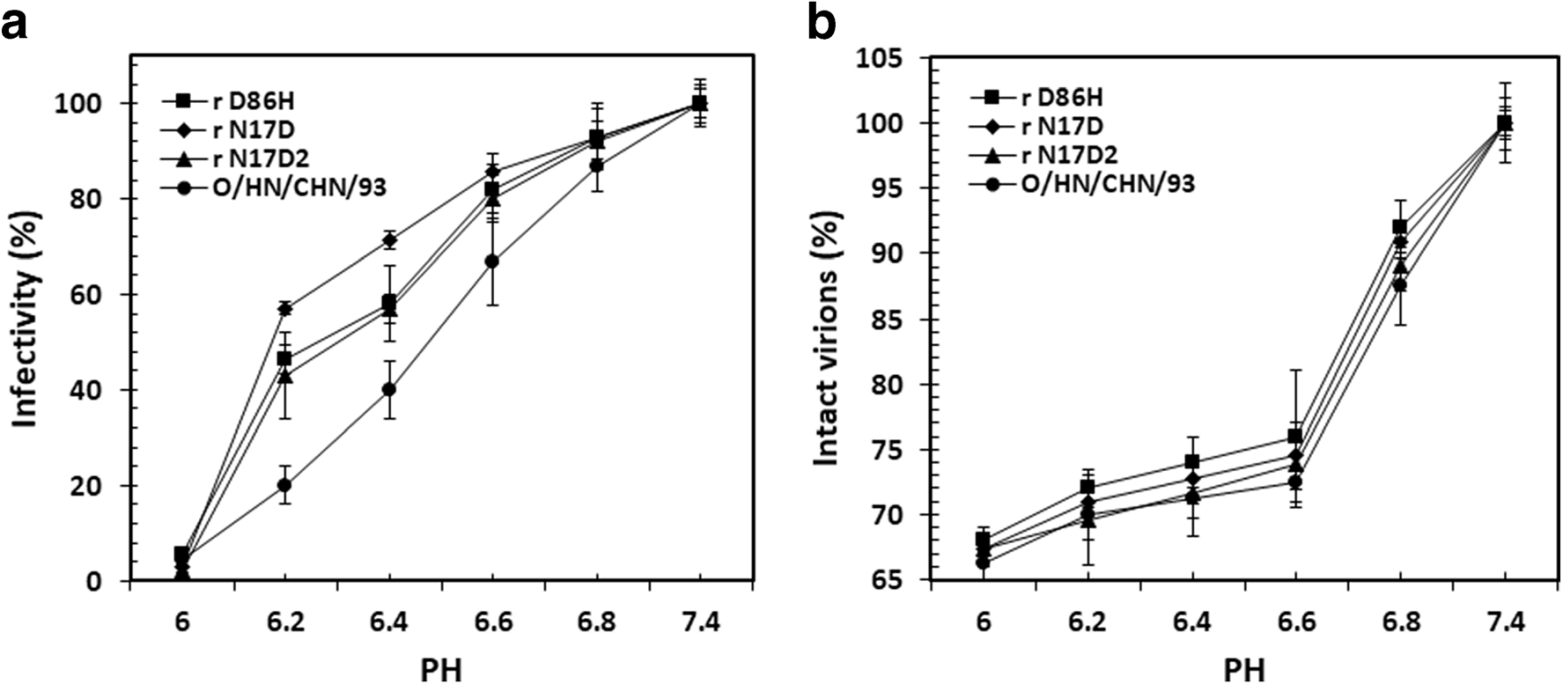
Acid sensitivity of the rescued FMDV. **a** Acid-induced inactivation analysis of the rescued FMDVs. Equal PFU amounts of the rescued viruses were treated with PBS at different pH values for 30 min at room temperature. The samples were neutralized with Tris at 1 M and pH 7.4 and the infectivity was expressed as the percentage of PFU recovered in BHK-21 cells relative to the obtained at pH 7.4. **b** Acid dissociation kinetic studies of FMDV r N17D, r D86H, r N17D2, and O/HN/93. Same amounts (PFU) of chemically inactivated (BEI) viruses tested were mixed with PBS of different pH values for 30 min at room temperature. Then, the solution was neutralized with 1 M Tris (pH 7.4) and the remaining 146S was determined by the size-exclusion high-performance liquid chromatography (SE-HPLC). The percentage of remaining 146S compared to those acquired at pH 7.4 was determined. Data were presented as means ± standard deviations (SD) of three independent experiments

To examine whether the rescued FMDVs increased resistance to acid inactivation, we compared the acid stability of the mutants and parental virus (Fig. 1a). Relative to the parental virus, the mutant viruses displayed acid-resistant phenotypes. The pH_50_ value for rD86H, rN17D, and rN17D2 was, respectively, 6.25, 6.18, and 6.30, while for the WT virus, the pH_50_ value was 6.50. Although the mutant rN17D2 had an acid-stable phenotype, the additional replacements in it seemed to counteract the acid resistance conferred by substitution VP1 N17D. These results confirmed that amino acid substitution VP1 N17D or VP2 D86H significantly increased resistance to acid inactivation, respectively (VP1 N17D, *P* < 0.001; VP2 D86H, *P* < 0.001).

The acid-induced inactivation of infectivity might be related to the dissociation of the virus capsid into its subunits. Therefore, the acid-induced disassembly assay was next performed at room temperature (Fig. 1b). As expected, the substitutions in rN17D, rD86H, and rN17D2 also increased resistance to acid-induced disassembly. However, it was observed that the level of acid-induced inactivation was different from that of acid-induced disassembly at the same acidic pH, indicating that acid inactivation and acid dissociation of FMDV might involve in different processes. At pH 6.0, the virions were observed to almost lost their infectivity, but nearly 65% percent of virion particles retained their intact capsids, which implied that some intact virions might lose their ability to infect cells in the acidic environment.

### Biological characteristics of the acid-resistant FMDV mutants

**Fig. 2:**
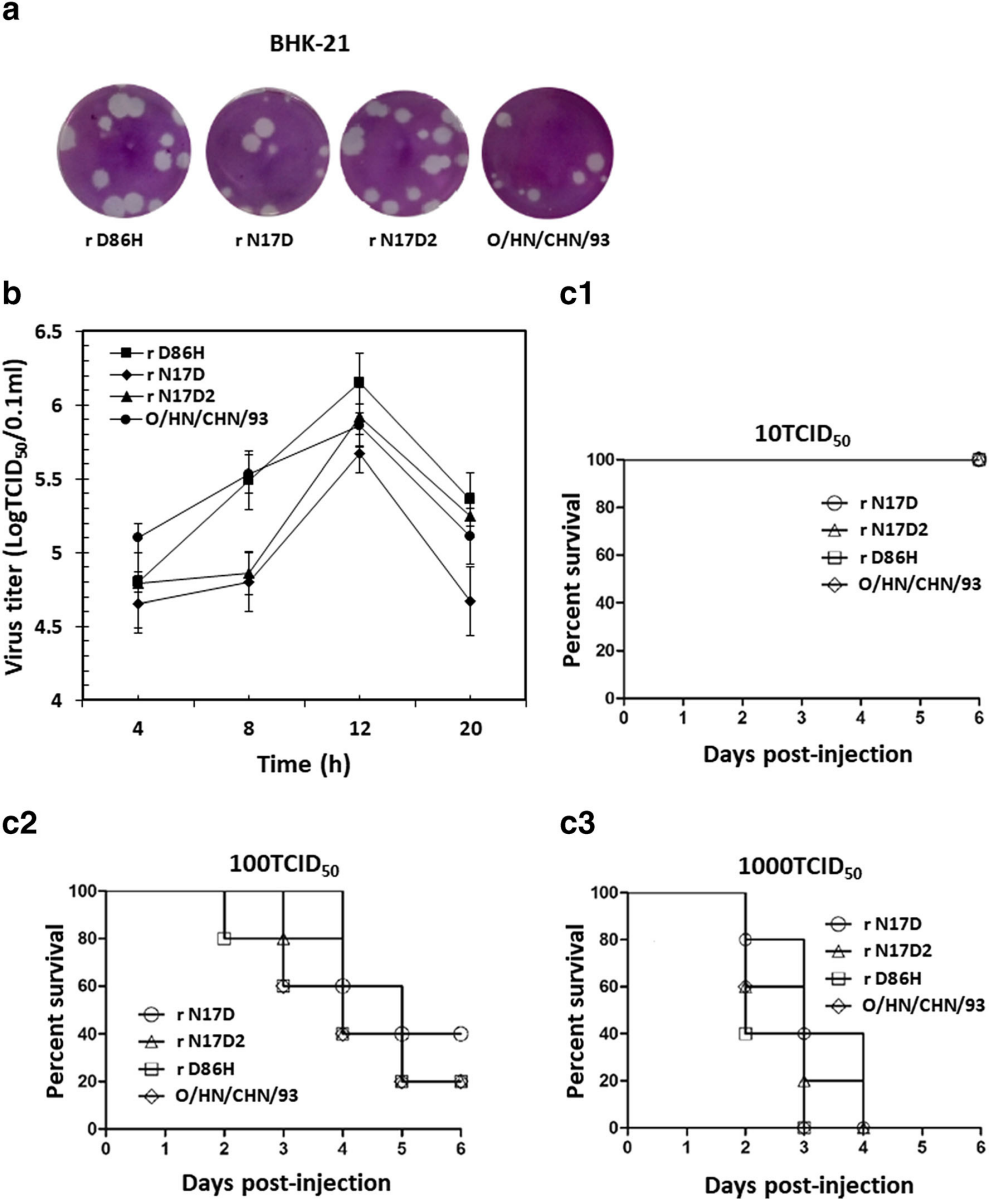
Biological characteristics of modified FMDVs with increased acid stability. **a** Plaque morphologies of the FMDV mutants and parental virus in BHK-21 monolayer cells. BHK-21 cells were infected with FMDVs and stained with crystal violet at 48 h post-infection (hpi). **b** One-step growth curves for the mutant viruses and parental virus in BHK-21 cells. BHK-21 cells were infected with FMDV mutants and parental virus at a multiplicity of infection (MOI) of 1 and the virus titers of harvested samples at different times (4 h, 8 h, 12 h, and 20 h) were determined by the plaque assay. **c** Virulence of the variants and its parental virus in suckling mice. One-day-old mice (5 per group) were inoculated with different 50% tissue culture infective dose (TCID_50_) of the modified viruses and parental virus and observed for 7 days (**c1**–**c3**). The percentage of animals surviving was calculated. Survivors were euthanized at the end of experiment

As shown in Fig. 2a, the three mutants showed similar plaque phenotype compared to the parental virus. The one-step growth curve of rescued viruses and parental virus in BHK-21 cells was determined (Fig. 2b). The result revealed that virus rD86H or rN17D2 grew with similar kinetics related to the parent virus; however, virus rN17D showed a slightly low level of titer as compared with the parental virus. Interestingly, the mutant rN17D2 always exhibited a higher titration than rN17D, possibly due to a compensating effect of the four additional mutations on reduced replication ability induced by substitution VP1 N17D.

The effect of substitutions responsible for the enhanced acid stability on FMDV virulence was analyzed in suckling mice. No suckling mice died in the mutant or parental groups inoculated with a dose of 10 TCID_50_ (Fig. 2c1), while all mice died in groups inoculated with a dose of 1000 TCID_50_ (Fig. 2c3). When inoculated with a dose of 100 TCID_50_ (Fig. 2c2), the final survival percentage varied from FMDV strains. The final survival rate of mice infected with the mutant r N17D was the highest, implying that substitution VP1 N17D resulted in mildly reduced virulence in suckling mice, which was consistent with the previous study that substitution VP1 N17D in virus O/YS/CHA/05 led to marginally decreased virulence in BALB/c suckling mice (Liang et al. 2014). For mutant rN17D2, the additional replacements might restore the decreased virulence caused by substitution VP1 N17D. Similar virulence was observed with r D86H virus and nonmutated virus. No suckling mice died in the control groups inoculated with PBS.

### Several biophysical stability analyses of FMDV mutants with improved acid stability

**Fig. 3:**
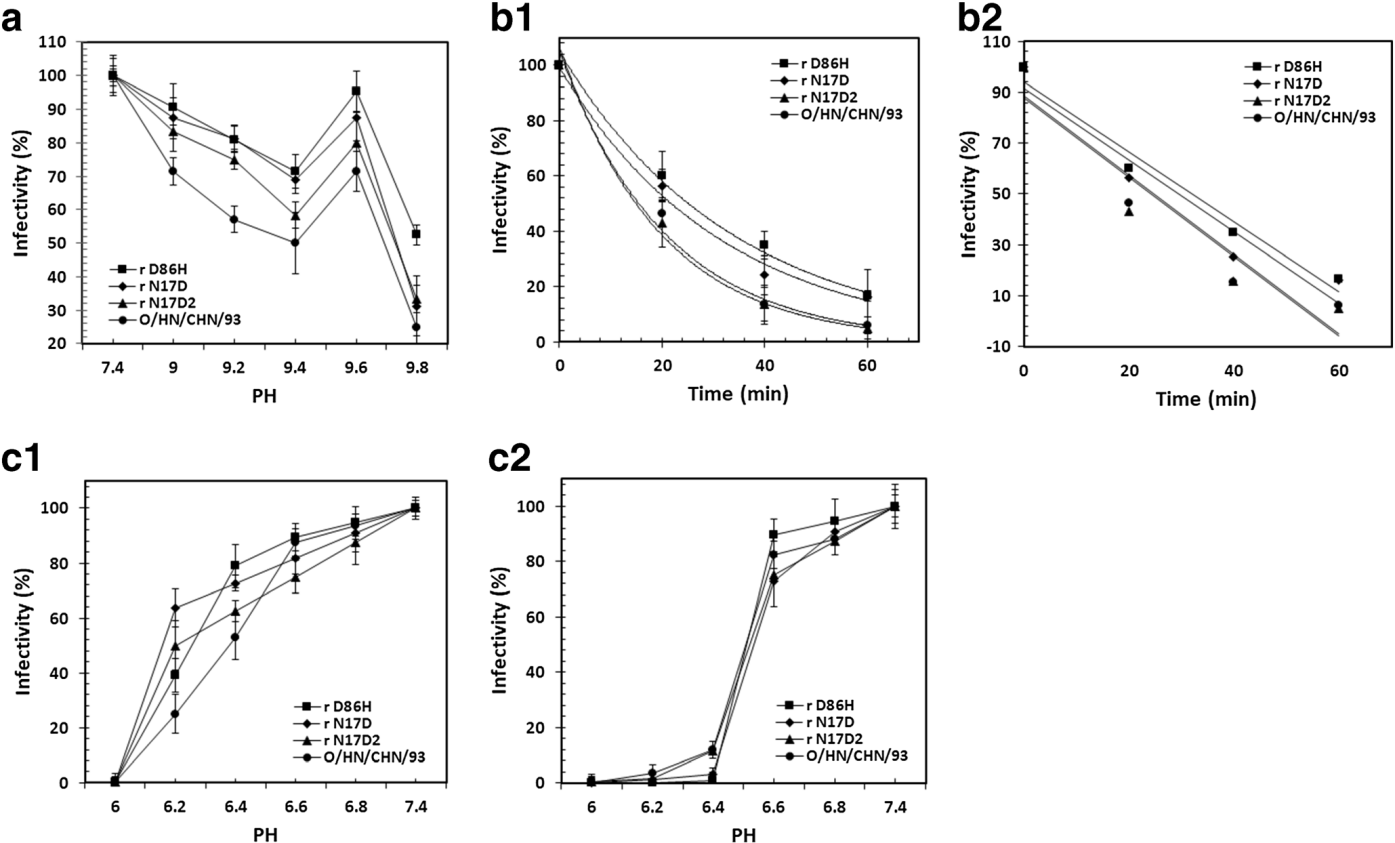
Several biophysical stability analyses of the acid-resistant FMDV mutants (**a**–**c**). **a** Alkali-induced inactivation kinetics of the acid-resistant FMDV mutants. Equal amounts of engineered FMD virions were treated with PBS solution of different pH values. The samples were then neutralized and added to BHK-21 monolayers. The infectivity was calculated as described above. **b** Thermal-inactivation kinetics of r N17D, r D86H, r N17D2, and O/HN/93 viruses. The different viruses were heated at 42 °C for up to 1 h. Then, the remaining virus in each case was determined in plaque assays and the infectivity was calculated (**b1**). All data were fitted to single-order exponential processes (**b2**). **c** Effect of acid treatment on the inactivation of FMD virions at different ionic strengths. The viruses were incubated with PBS buffer (ranging from pH 6.0 to 7.4) and NaCl solutions (150 mM or 1 M) for 30 min. After neutralization, the recovered virus was determined and the infectivity was calculated

In the practical production, alkalis such as 2% sodium hydroxide and 4% sodium carbonate can disinfect against FMDV (Owen 1995). To date, little is known about the influence of alkali treatment on the stability of acid-resistant FMDV. The alkali-induced inactivation assay indicated that amino acid substitutions responsible for the enhanced acid stability resulted in moderate increase in the alkali stability (Fig. 3a1), implying that the similar mechanism between acid stability and alkali stability was involved. Interestingly, the amount of infectious FMDVs at pH 9.6 was higher than that at pH 9.4 and pH 9.8, suggesting that the alkali-induced inactivation was not achieved immediately and the alkali stability was regulated by multiple factors which were related to complex interactions between residues in capsid proteins.

FMD virions are not only remarkably acid labile but also extremely thermolabile. Mild heating induces dissociation of capsids into subunits, resulting in the acute loss of immunogenicity (Mateo et al. 2008; Rincon et al. 2014). It has been reported that single amino acid substitution with increased stability against acid-induced inactivation can also confer increased resistance to heat inactivation (Martin-Acebes et al. 2011). In order to obtain more stable FMDV, we then examined the effects of introduced mutations on the inactivation rate of mutant virions at 42 °C (Fig. 3b1 and b2). Our experiments confirmed a minor increase in the resistance to thermal inactivation for acid stable r D86H and r N17D mutants. In contrast, the mutant rN17D2 inactivated at the same rate as that of the parental virus at 42 °C, showing that the additional replacements in rN17D2 reduced thermostability against inactivation conferred by substitution VP1 N17D.

The effect of acid treatment at different ionic strengths on the stability of mutant viruses was also determined in the presence of NaCl at concentrations of 150 mM (Fig. 3c1) or 1 M (Fig. 3c2) (Maree et al. 2013; Rincon et al. 2014). At a concentration of 150 mM NaCl, the infectivity of FMDV mutants was reduced by the treatment with acidic pH, which was similar to the result of acid-induced inactivation. As expected, at a concentration of 1 M NaCl, a dramatic decrease in virus infectivity was observed for the mutants and parental virus at pH 6.4, which was consistent with the previous observations showing that high ionic strength may destabilize the capsids (Curry et al. 1996). The result also showed that rD86H and rN17D mutants were inactivated slightly faster than the parental virus at high NaCl concentration, and rN17D2 mutant was almost inactivated at the same rate as that of the parental virus.

### Structure analysis and molecular modeling

**Fig. 4:**
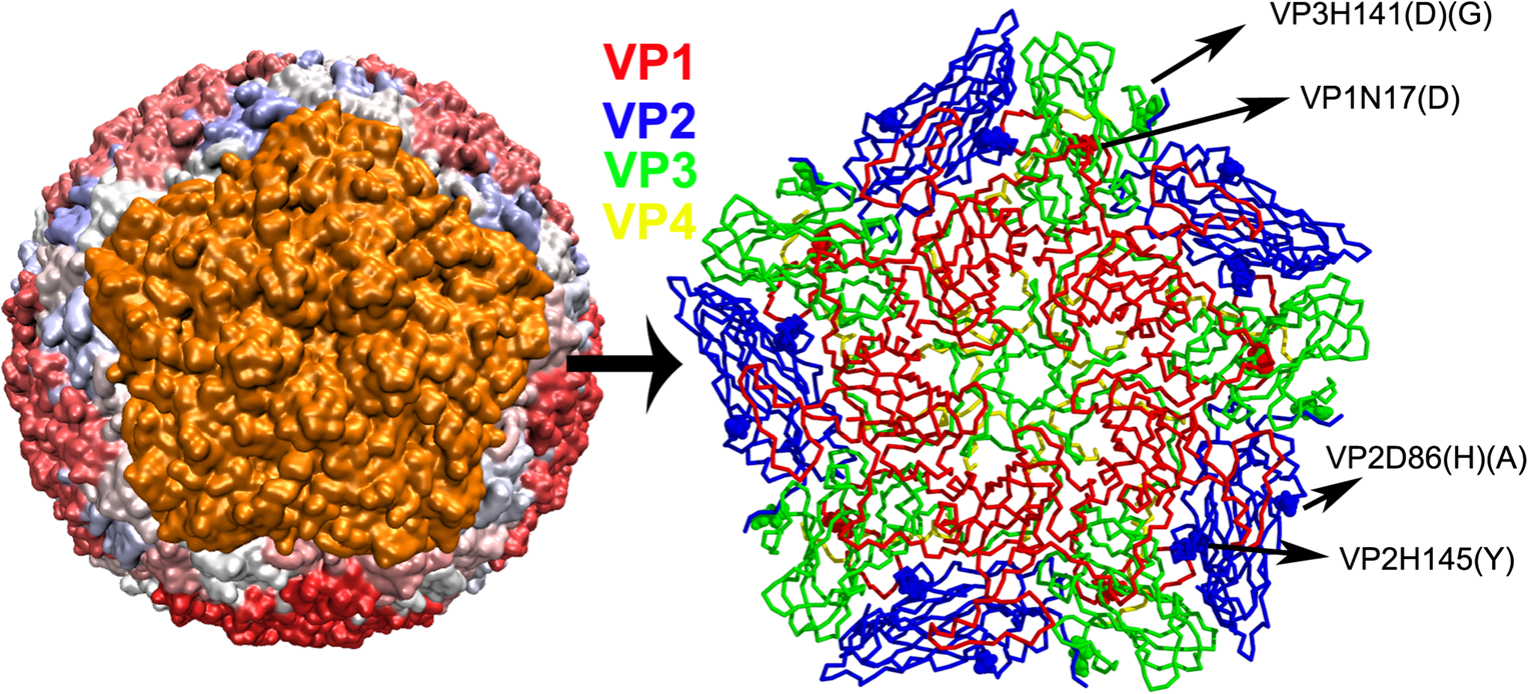
The structural model of FMDV. The left sphere is the capsid model of FMDV, and the right cartoon representation is the pentamer containing VP1, VP2, VP3, and VP4. The mutated residues are marked on the pentamer

**Fig. 5:**
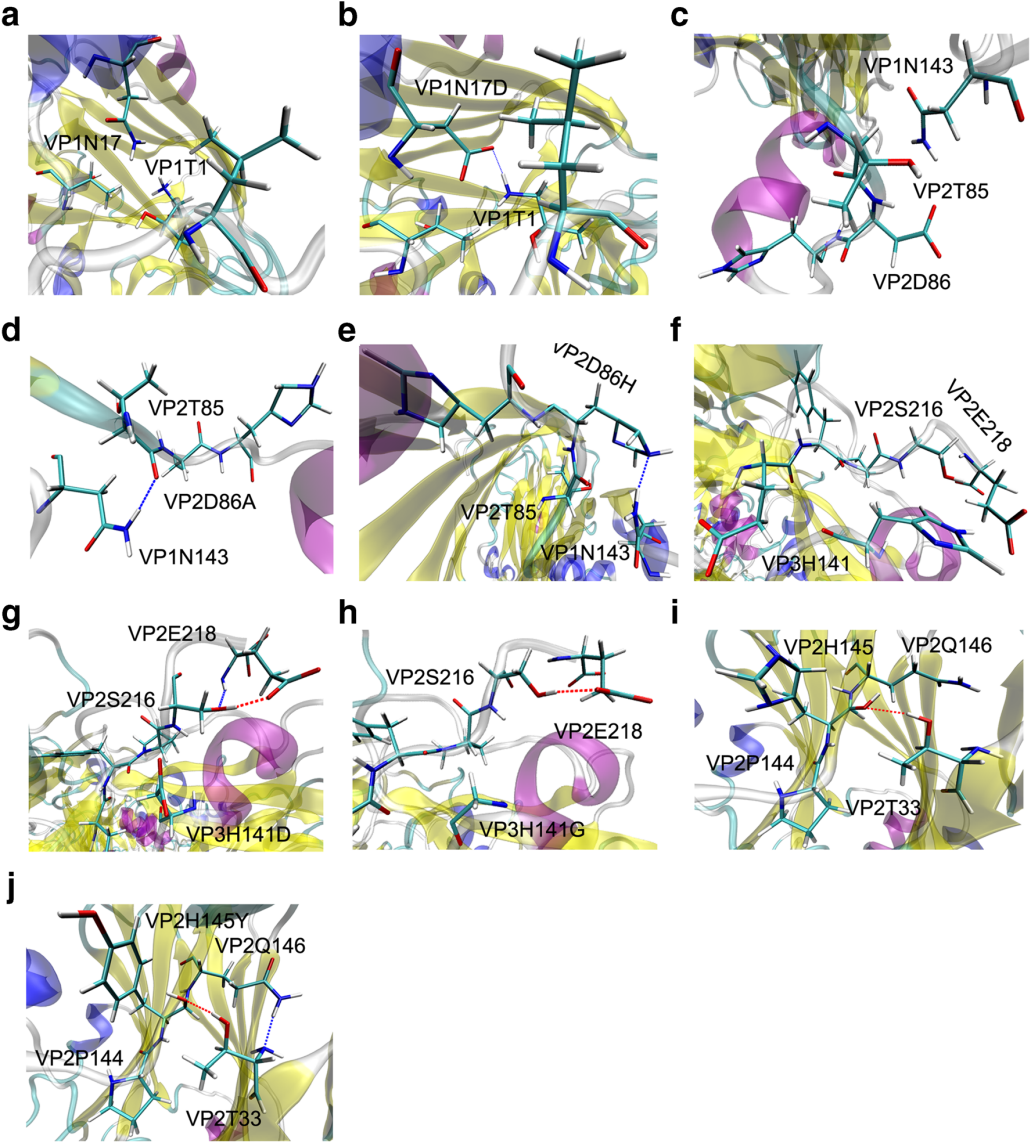
The hydrogen bond analysis. **a**–**j** The hydrogen bonds between the former or mutated residues (VP1N17D, VP2H145Y, VP2D86H, VP2D86A, VP3H141G, and VP3H141D) and other residues in FMDV capsid. The red and blue lines represent the hydrogen bonds

The complete, accurate model of FMDV can enhance the understanding of capsid structural properties at the atomic level. As shown in Fig. 4, the full model of FMDV was formed by 12 pentamers. To model the stability of FMDV with site-specific mutagenesis, the mutation VP1 N17D, VP2 H145Y, VP2 D86H, VP2 D86A, VP3 H141G, and VP3 H141D were chosen to analyze hydrogen bonds by comparisons with our virus experiments. After the VP1 N17D mutation, VP1 T1 formed a hydrogen bond with mutated residue VP1 N17D (Fig. 5a, b), strengthening the stability of the capsid. Due to the reduced conformation occupation of VP2 D86A, VP2 T85 had free space to form a hydrogen bond with VP1 N143 (Fig. 5c, d). In contrast, VP2 D86H had sufficient space for rotation to form a hydrogen bond with VP1 N143 (Fig. 5c, e); thus, the virus with the VP2 D86H substitution formed stronger hydrogen bonding compared to the one with the VP2 D86A substitution. In the VP3 H141D and VP3 H141G mutants, there was more space for hydrogen bonding between VP2 S216 and VP2 E218 because of the substitution of the imidazole functional group of His (Fig. 5f–h). Besides, the VP2 H145Y mutation had a stronger hydrogen bond between VP2 T33 and VP2 Q146 than that of VP2 H145 (Fig. 5i, j). These results indicated that all substitutions were inclined to form new hydrogen bonds to keep the FMDV structure stable, which corresponded with the stability assays.

### The immunogenicity of acid stable FMDV mutants in guinea pigs

For the potential use in vaccine improvement, the immunogenicity of acid stable FMDV mutants was evaluated in guinea pigs using virus neutralization (VN) assay. As shown in Table 1, no significant differences were found between the neutralizing antibody responses elicited by three FMDV mutants and parental virus antigens. This result revealed that substation VP1 N17D or VP2 D86H had no detectable effect on the immunogenicity of FMDV O/HN/CHN/93.

**Table 1:** Immunogenicity comparison between the acid stable FMDV mutants and parental virus

Days post-vaccination	Neutralizing antibody titer (NT_50_)				
	Anti-O/HN/CHN/93	Anti-rD86H	Anti-rN17D	Anti-rN17D2	Anti-PBS
**0**	**< 4**	**< 4**	**< 4**	**< 4**	**< 4**
**14**	**32**	**32**	**32**	**32**	**< 4**
**21**	**79.6**	**70.6**	**66.8**	**66.8**	**< 4**
**28**	**90**	**90**	**84.8**	**84.8**	**< 4**
**35**	**120.4**	**105.2**	**105.2**	**112.8**	**< 4**

The sera were collected from five guinea pigs in each group. Neutralizing antibody titer (NT_50_) was expressed as the highest serum dilution to neutralize 100 TCID_50_ FMDV in 50% of the wells. The NT_50_ determinations were repeated twice and presented as an average value

## Discussion

### Introduction of amino acid substitutions to improve the acid stability of engineered FMDV

Diverse amino acid substitutions responsible for the acid sensitivity phenotype of FMDV have been reported recently (Xie et al. 2019; Yuan et al. 2017). Based on those previous findings, to develop acid-stable inactivated vaccines, a panel of substitutions (VP1 N17D, VP2 H145Y, VP2 D86H, VP3 H141D, VP3 H141G, and VP1 N17D/VP2 H145Y) were introduced into infectious cDNA clone pOFS which contained the complete sequence of O/HN/CHN/93. The nearly isosteric substitution VP1 N17D, which is located at the internal region of capsid and close to but not at the inter-pentamer interfaces, has been screened out among different FMDV serotypes. Our result showed that O/HN/CHN/93 carrying this substitution had an acid-, alkaline-, and thermal-stable phenotype, but displayed a reduced replication ability and slightly deferred virulence in suckling mice. The VP2 H145Y substitution selected in serotype Asia1 and C FMDV is also mapped to the internal region of the capsid at the intra-protomeric interface, affecting the protomer structure and pentamer stability (Vazquez-Calvo et al. 2014; Wang et al. 2014). It has been proposed that residue VP2 H145 is critical to the cleavage of VP0 and its substitution is lethal in poliovirus (Hindiyeh et al. 1999). In this study, we could not recover the FMDV mutant carrying the single replacement VP2 H145Y or double replacements VP2 H145Y + VP1 N17D in the background of parental O/HN/CHN/93 owing to reverse and additional mutations. This suggested that substantial increase in the acid stability of FMDV was not easy to occur and residue VP2 H145 may play important role in virus functions. In addition, the additional mutations in rN17D2, which were caused by the reverse mutation of substitution VP2 H145Y, might compensate for functions conferred by substitution VP1 N17D. Residue VP3 H141 situated at the pentamer–pentamer interface has been considered as a huge destabilizing factor for FMDV capsid and a likely trigger for uncoating (Curry et al. 1995; van Vlijmen et al. 1998). As noted by Ellard et al., the replacement VP3 H141D indeed improved the FMDV stability under acidic conditions (Ellard et al. 1999). However, our research indicated that it was impracticable for the O/HN/CHN/93 strain that residue VP3 H141 was substituted to a residue with negative charge or with smaller side chain. Substitution VP2 D86H has been selected by our laboratory in type O FMDV acid-resistant mutants (data not shown). The structure modeling showed that residue VP2 D86 was located at inter-pentamer interfaces around the twofold axes of symmetry which played a crucial role in controlling FMDV capsid stability. Our study proved that the FMDV mutant carrying substitution VP2 D86H was more acid, alkaline, and thermal stable without impairing its infectivity in comparison with the parental virus. In conclusion, we successfully rescued three acid stable FMDV mutants (rN17D, rD86H, and rN17D2) with differences in biological characteristics.

### The analysis of biophysical stability of FMDV mutants

In recent years, physics and chemistry are being increasingly recognized as important tools to qualitatively and quantitatively investigate viruses (Adams et al. 2005). During the manufacturing process of inactivated FMD vaccines, a variety of factors, such as temperature, pH value, and ionic strength, have a great effect on FMDV stability. Here, we also determined other biophysical stability of FMDV mutants with enhanced acid stability. The pH stability of FMDV capsid is involved in acid sensitivity and alkali sensitivity. FMDV acid sensitivity is mainly regulated by electrostatic interactions and hydrogen bonds formed by the side chain of amino acid residues in the capsid, while little is known about FMDV alkaline stability. We examined the remaining infectivity of acid stable FMDV after exposure to the alkaline pH values. A moderated increase in the alkaline resistance for the mutant rN17D or rD86H was revealed compared with the parental virus. To our surprise, the level of virus yield increased at pH 9.6 compared with that at pH 9.4, unlike that the level of infectious virus gradually reduced as the pH dropped. This suggested that the mechanism of acid stability may not be the same as that of alkaline stability. FMDV thermal stability can be influenced by the disulphide bridges, electrostatic interactions, hydrogen bonds, and salt bridges (Kotecha et al. 2015; Loladze et al. 1999; Rincon et al. 2014). Because there are some similarities in the determining factors of FMDV acid stability and thermostability, it should be possible that an acid-resistant FMDV can increase its resistance to thermal inactivation. As expected, the FMDV mutant rN17D or rD86H showed increased resistance to either acid- or thermal-induced inactivation. It has been reported that a high ionic strength would screen surface charges and reduce coulombic interactions of pentamers, destabilizing FMDV capsids and facilitating the loss of virus infectivity (Curry et al. 1996; Maree et al. 2013; Rincon et al. 2014). Consistently, at acidic pH, the high ionic strength was observed to significantly inactivate the FMDV particles. Specially, when compared with the parental virus, the mutant rN17D and rD86H seemed more likely to be inactivated at pH 6.4, possibly because that the neutral-to-charged VP1 N17D substitution and negatively-to-positively charged VP2 D86H substitution in the two FMDV mutants introduced electrostatic repulsions at high ionic strength, which facilitated capsid dissociation. To sum up, the single amino acid substitution VP1 N17D or VP2 D86H could increase the acid, alkaline, and thermal resistance, but slightly impaired the FMDV stability in high ionic strengths.

### Improvement of FMD vaccines with acid stability

As the acid lability is the requirement of FMDV uncoating, the continuous increase in acid stability is selectively constrained without functional impairment for infectivity. So far, nearly 11 amino acid substitutions have been confirmed to confer resistance to acid-induced FMDV inactivation (Yuan et al. 2017). Among them, substitution VP1 N17D is widely found to be responsible for acid-resistant phenotype of FMDV and seems to have great potential to be used in the improved FMD vaccines with acid stability. However, for strain O/HN/CHN/93, the introduction of substitution VP1 N17D influenced viral infectivity. Therefore, it is difficult to obtain more robust FMDV mutants without disrupting viral infectivity. A step forward would be the combination of artificial selection, structure model, and reverse genetics technique.

In addition to the acid lability, FMD virions are remarkably thermolabile, which leads to the dramatic losses of immunogenicity. Therefore, vaccine integrity relies heavily on expensive, difficult-to-maintain cold chain from manufacture to use, and this is often unfeasible in endemic regions or in field conditions (Kotecha et al. 2016; Mateo et al. 2007; Mateo et al. 2008; Rincon et al. 2014). Thus, both the enhanced acid stability and the increase in the resistance against thermal dissociation of virus capsid are crucial for the vaccine efficacy during the process of vaccine manufacturing. In this work, the genetically modified FMDV rN17D or rD86H was proved to present acid- and thermal-resistant phenotype compared with its parental virus O/HN/CHN/93. Furthermore, the infectivity was not impaired for the mutant rD86H. In summary, the mutant r D86H obtained could provide the basis for improving the biophysical stability of conventional vaccines based on inactivated virions. Moreover, the substitution VP2 D86H could be introduced into empty capsid to improve the stability of empty capsid vaccines. We expect that the development of chemically stable vaccines may provide powerful weapons for the FMD control and eradication.
